# TILLING to detect induced mutations in soybean

**DOI:** 10.1186/1471-2229-8-9

**Published:** 2008-01-24

**Authors:** Jennifer L Cooper, Bradley J Till, Robert G Laport, Margaret C Darlow, Justin M Kleffner, Aziz Jamai, Tarik El-Mellouki, Shiming Liu, Rae Ritchie, Niels Nielsen, Kristin D Bilyeu, Khalid Meksem, Luca Comai, Steven Henikoff

**Affiliations:** 1 Fred Hutchinson Cancer Research Center, Seattle, WA 98107, USA; 2 Department of Biology, University of Washington, Box 355325, Seattle, WA 98195, USA; 3 National Center for Soybean Biotechnology, Division of Plant Sciences, University of Missouri, Columbia, MO 65211, USA; 4 Department of Plant Soil and Agricultural Systems, Southern Illinois University, Carbondale, IL 62901, USA; 5 USDA-ARS Crop Production and Pest Control Research Unit, Purdue University, West Lafayette, IN 47907, USA; 6 USDA-ARS Plant Genetics Research Unit, Columbia, MO 65211, USA; 7 Current address: Department of Plant Biology and Genome Center, UC Davis, Davis, CA 95616, USA

## Abstract

**Background:**

Soybean (*Glycine max *L. Merr.) is an important nitrogen-fixing crop that provides much of the world's protein and oil. However, the available tools for investigation of soybean gene function are limited. Nevertheless, chemical mutagenesis can be applied to soybean followed by screening for mutations in a target of interest using a strategy known as Targeting Induced Local Lesions IN Genomes (TILLING). We have applied TILLING to four mutagenized soybean populations, three of which were treated with ethyl methanesulfonate (EMS) and one with N-nitroso-N-methylurea (NMU).

**Results:**

We screened seven targets in each population and discovered a total of 116 induced mutations. The NMU-treated population and one EMS mutagenized population had similar mutation density (~1/140 kb), while another EMS population had a mutation density of ~1/250 kb. The remaining population had a mutation density of ~1/550 kb. Because of soybean's polyploid history, PCR amplification of multiple targets could impede mutation discovery. Indeed, one set of primers tested in this study amplified more than a single target and produced low quality data. To address this problem, we removed an extraneous target by pretreating genomic DNA with a restriction enzyme. Digestion of the template eliminated amplification of the extraneous target and allowed the identification of four additional mutant alleles compared to untreated template.

**Conclusion:**

The development of four independent populations with considerable mutation density, together with an additional method for screening closely related targets, indicates that soybean is a suitable organism for high-throughput mutation discovery even with its extensively duplicated genome.

## Background

Much of the world's protein and oil comes from soybean (*Glycine max *L. Merr.), and it is the major source of seed meal used in animal feed. In fact, soybean contains more protein than any other ordinary food source, including meat, cheese and fish [1]. It grows in a variety of temperate climates, and has the added benefit of improving soil quality by fixing nitrogen. Except for corn, more soybean is grown in the USA than any other single crop.

Unfortunately, despite the importance of soybean, genetic tools for investigation of gene function and crop improvement have been difficult to develop. Although soybean can be transformed with either *Agrobacterium tumefaciens *or *A. rhizogenes*, neither system is ideal. The efficiency of *A. tumefaciens *transformation is typically low [2,3] and is genotype specific [4]. Currently, the most successful combination of genotypes, chemical enhancers and selection, yields transformation efficiencies of up to 16% [5]. *A. rhizogenes *root transformation has higher efficiency (about 50–90%) and seems to be genotype independent, but is not heritable [6,7]. Particle bombardment can also be used to obtain transformants with variable success rates [8,9], but can also introduce multiple copies that may recombine or result in co-suppression [10]. Often the goal is to obtain a knockout to better understand gene function. However, gene disruption by induction of transposon insertion has not yet been successful. RNAi has produced knockdowns in some cases [11,12], but still relies on transformation. Additionally, all of these methods require time-consuming tissue culture steps that are not compatible with high-throughput generation of mutants, and still can produce chimeric transformants that may not pass the trait on to the next generation.

In contrast to transgenic methods, chemical mutagenesis can be applied to most species, even those that lack well-developed genetic tools. Chemical mutagenesis has several other benefits. No tissue culture is required, and the induced changes are stable and heritable so that the succeeding generations will not be chimeric. Because chemical mutagenesis induces single nucleotide changes, it can provide an allelic series in a gene target in addition to knockouts. Importantly, lines carrying induced mutations are not transgenic, and are therefore not associated with any regulatory restrictions. Chemical mutagenesis has been successfully used for many phenotypic screens in soybean, yielding mutants in traits such as ethylene sensitivity and nodulation [13,14]. The combination of chemical mutagenesis with screening for induced changes in a gene target of interest is a powerful technique for obtaining an allelic series that can be used to study gene function or crop improvement.

**Figure 1:**
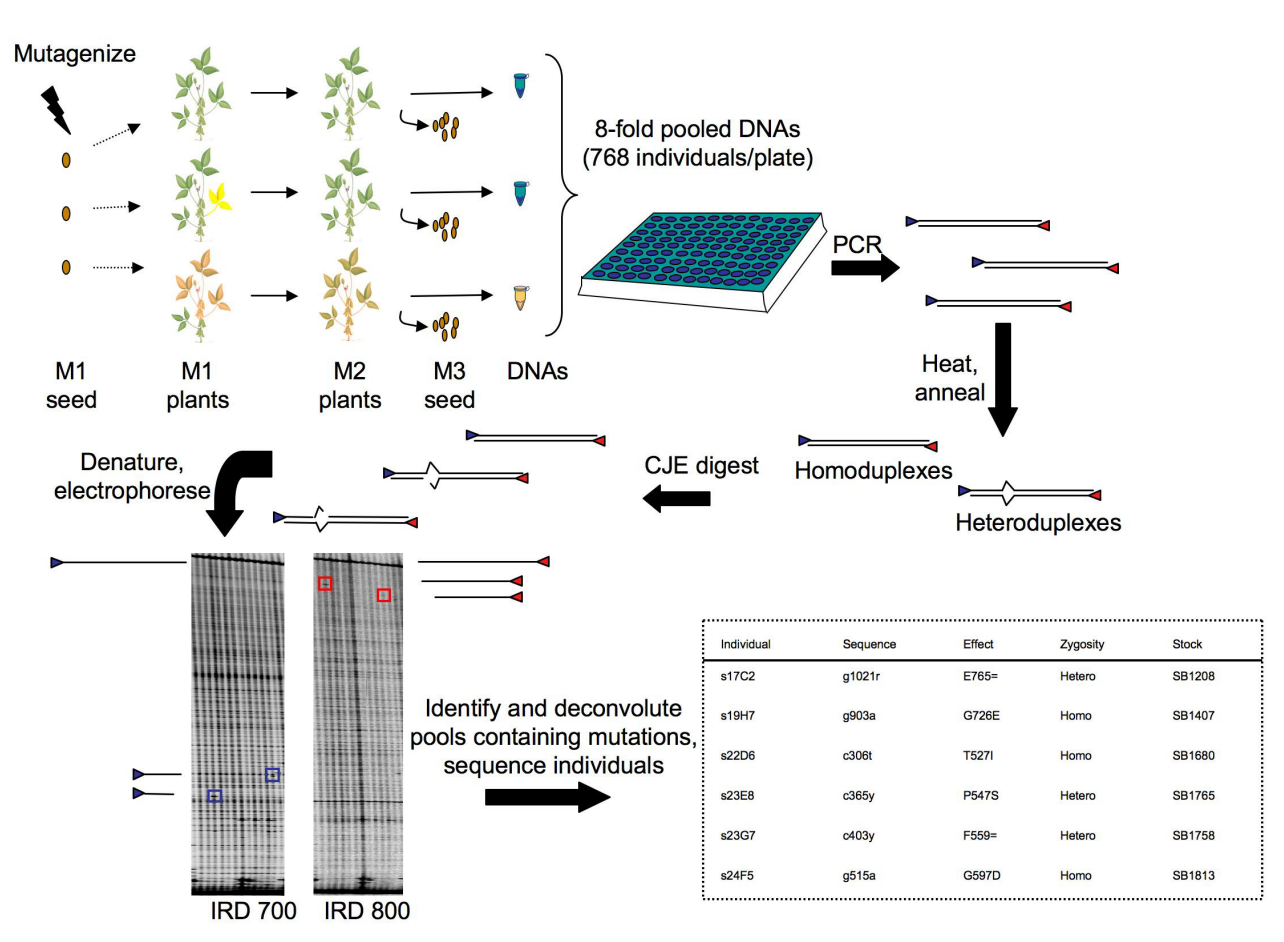
Schematic of the soybean TILLING process [39]. Seeds are mutagenized and grown to generate the M1. Since the embryo consists of many cells, M1s may be mosaic for mutations induced by the mutagen. M1 plants are allowed to self and a single M2 plant is grown from each M1 line. Tissue and M3 seed are collected from the M2 plants. The concentration of DNAs isolated from the M2 tissue is normalized, and the samples are pooled eight-fold in 96-well plates. IRDye labeled primers are used for amplification of a particular target. Following PCR, samples are denatured and allowed to reanneal such that if a mutation is present, heteroduplexes will form. CJE is used to cleave 3' of the mismatch. Samples are denatured and electrophoresed on polyacrylamide gels using LI-COR 4200 or 4300 machines. Putative mutations are identified by bands appearing in the 700 and 800 channels that add up to the molecular weight of the full length PCR product. Pools are deconvoluted to identify mutant individuals, and the individuals are sequenced. Sample soybean gel section and complete results from the gmclavb primer set screened on the A population are shown.

TILLING (Targeting Induced Local Lesions IN Genomes) is a high-throughput reverse genetic method to obtain allelic series from a chemically mutagenized population (Figure 1). A chosen target is amplified from pooled DNAs using fluorescently labeled PCR primers. Following amplification, the PCR products are denatured and re-annealed. If a mutation is present in the pooled DNA, a heteroduplex will form. A single-strand specific nuclease found in celery juice extract (CJE) is used to cleave a strand of the heteroduplex, and the products are electrophoresed on a denaturing acrylamide gel [15]. Mutations are detected by the observation of cleaved bands.

We have established a popular TILLING service for *Arabidopsis thaliana*, where we have identified over 6700 mutations in more than 570 targets during the past five years [16]. TILLING has also been successfully applied to maize, barley and wheat, despite their having much larger genomes than Arabidopsis [17-19]. Here we extend TILLING to four chemically mutagenized soybean populations and describe a generally applicable strategy for eliminating amplification of multiple products from the closely related homeologs or paralogs in the soybean genome.

## Results

### Mutation discovery in mutagenized soybean populations

Four mutagenized soybean populations in two genetic backgrounds were constructed for TILLING, referred to as A, B, C, and D (Table 1). The chemical mutagens NMU and EMS have been shown to induce mutations in previous phenotypic screens of soybean [13,14]. Genomic DNA was isolated from leaf tissue and samples were normalized prior to pooling eight-fold for screening. Each population was screened independently with the same primers (Table 2).

**Table 1:** Soybean TILLING populations.

Population	Size	Cultivar	Mutagen	Concentration
A	529	Forrest	EMS	40 mM
B	768	Williams 82	EMS	40 mM
C	768	Williams 82	EMS	50 mM
D	768	Williams 82	NMU	2.5 mM

**Table 2:** Primer sequences.

Primer	Sequence	GenBank
gmclav Left	5'-cgtggcaacgtgttcttcgttcag	AF197946
gmclav Right	5'-gtccggtgagattgttgccgctta	
gmclavb Left	5'-cgcagttccgtcagggattttcaa	AF197946
gmclavb Right	5'-ttgggtccaccactgccaacacta	
gmnark Left	5'-cttcttccgcggtccaatccctaa	AY166655
gmnark Right	5'-gcaatgtagccgtaggagccagca	
gmppck4 Left	5'-tgaagcaaaacccaaagctgtttgaga	AY568714
gmppck4 Right	5'-acccaacctccaagttgcgtttcttta	
gmrhg1b Left	5'-cctcgcttaggcagcttgatttgtca	AF506516
gmrhg1b Right	5'-tagcaactcgtcgccaactgtgga	
gmrhg4b Left	5'-gaagttggtgactgcgggaaatgc	AF506518
gmrhg4b Right	5'-ttcaatgcaccgatccaacaagga	
gmsacpd2 Left	5'-agagggcaaaggagcttccagatgatt	AY885234
gmsacpd2 Right	5'-ttgcttgagctctctcctccaaccttc	

**Figure 2:**
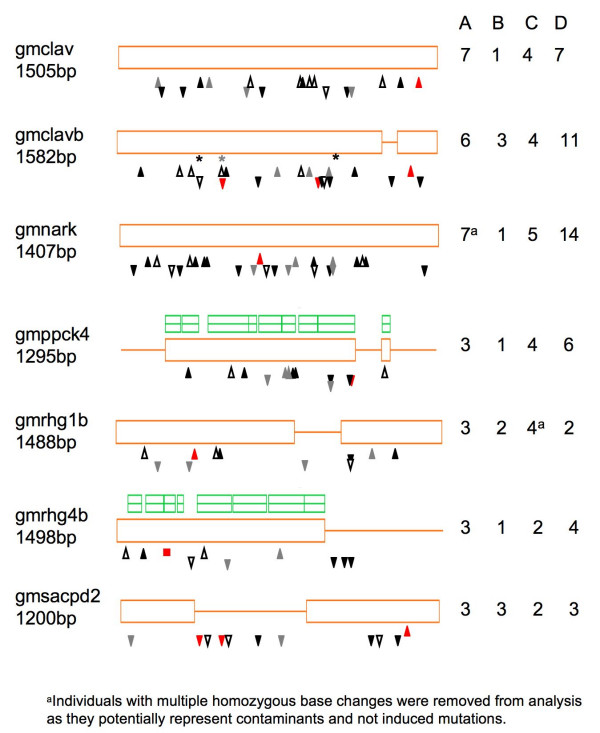
Type and distribution of induced mutations discovered in seven amplicons. Orange boxes correspond to exons, lines to introns. Homology to proteins in the BLOCKS database [38] is indicated by the green boxes above gmppck4 and gmrhg4b. The other amplicons did not contain regions of BLOCKS homology. Arrowheads indicate approximate position of missense changes, upside down arrowheads indicate silent changes, asterisks indicate nonsense mutations, boxes indicate deletions. Hollow arrowheads = A population; red = B population; gray = C population; black = D population. The number of mutations discovered in each amplicon per population is indicated on the right.

We discovered 116 mutations: 32 in A, 12 in B, 25 in C, and 47 in D (Figure 2 and Additional File 1). Two individual lines, one from the A population and the other from C, had more than one base change detected in an amplicon. Because these changes were homozygous and not the expected G/C to A/T EMS-induced transitions, we considered the individual lines to be likely cultivar contaminants, and we excluded them from the analysis. Mutation density was estimated as the total number of mutations divided by the total number of base pairs screened (amplicon size × individuals screened). For each target, 200 bp is subtracted from the amplicon size to adjust for the 100 bp regions at the top and bottom of TILLING gel images that are difficult to analyze [20]. The A and D populations showed similar mutation densities (~1/140 kb for both). Mutation density in the population designated C was ~1/250 kb and ~1/550 kb in the B population.

The C and D populations had the same distribution of mutations with 4% truncation mutations, 44–45% missense, and 51–52% silent mutations. The distribution in the B population was 8% truncation, 33% missense, and 58% silent mutations. The A population deviated significantly from these mutation distributions in that no truncations were found, 66% missense and 34% silent mutations were found (pairwise comparison of mutation distribution in A to distribution in each population: B χ2 = 15.5, p < 0.001; C χ2 = 6.62, p < 0.05; D χ2 = 6.05, p < 0.05). However, none of the distributions of mutations were significantly different than the expected distribution calculated from EMS-induced changes in the targets (3% truncations, 50% missense, and 48% silent).

In the A and C EMS-treated populations, as well as the NMU-treated D population, ~90% of base changes were G/C to A/T transitions (Table 3). In the EMS-treated B population, 75% of base changes were G/C to A/T transitions. However, the frequency of G/C to A/T transitions is not statistically significantly different between the B population and the other three populations. Each EMS-treated population contained an individual with a T to A transversion. The NMU population contained 3 individuals with G to T transversions. Because it is well established that EMS mutagenesis induces G/C to A/T transitions, the most conservative estimation of mutation density would only consider such base changes to be induced mutations. In that case, the mutation densities become ~1/200 kb in A, ~1/800 kb in B, and ~1/300 kb in C.

**Table 3:** Spectrum of mutations sequenced from seven targets in common among four populations.

**Population**	**g-->a**	**c-->t**	**g-->t**	**c-->a**	**g-->c**	**t-->c**	**a-->c**	**t-->a**	**a-->t**	**deletion**
**A**	15	15	0	0	0	0	1	1	0	0
**B**	8	1	0	0	0	0	0	1	1	1
**C**	15	8	0	0	0	1	0	1	0	0
**D**	20	22	3	1	1	0	0	0	0	0

### Elimination of near-duplicate amplicons

Three primer sets were initially tested for amplification of a specific target by observation of a single band of the expected size on an agarose gel. Although all three primer sets yielded a single band on an agarose gel, only one set (gmnark) produced good quality TILLING gels as determined by adequate quantities of single stranded full-length PCR product and by the detection of a low number of cleaved bands likely to represent induced mutations based on expected densities of chemically induced mutations in plants. Amplification products from the other two primer sets resulted in TILLING gels with multiple cleaved fragments in every lane, suggesting that more than one target was being amplified and digested.

**Figure 3:**
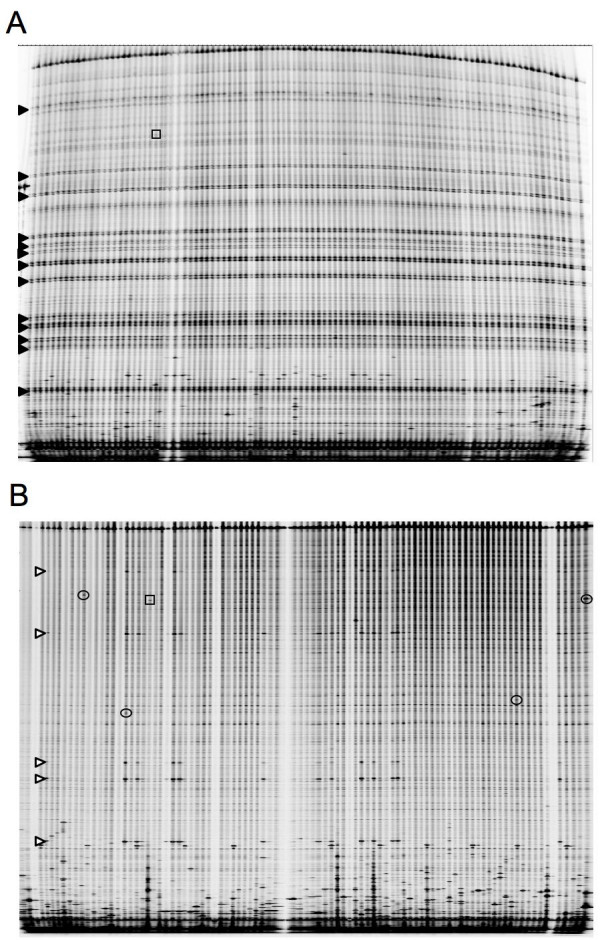
Elimination of multiple amplicons. Only the 700 channel is shown. Box indicates a cut DNA strand corresponding to a single nucleotide polymorphism that was identified and sequenced from both undigested and digested templates. A) Filled arrowheads indicate multiple bands in every lane of an eight-fold pool plate. These spurious cut products were derived from CJE digestion of heteroduplexes formed between PCR products from co-amplified targets, presumably homeologs. B) The same template pools from (A) digested with *ApaI *prior to PCR amplification for TILLING. Ovals denote cut DNA strands corresponding to single nucleotide polymorphisms that were identified only when TILLING from digested template. Open arrowheads show the position of bands from CJE digestion that represent polymorphisms present in more than one member of the population.

Following this observation, subsequent primers were tested by agarose gel analysis and sequencing. Of 27 primer sets tested, 17 primer sets amplified more than one target. Given the high proportion of tested primer sets that amplified more than one target, we wondered whether we could screen for mutations in these targets by eliminating extra templates in the genomic DNA. For example, amplification and CJE digestion with the gmrhg4 primers resulted in multiple bands in every pool (arrowheads, Figure 3A). The multiple bands were still observed when TILLING assays were performed on unpooled DNAs (data not shown), and multiple heterozygous sites were detected upon sequencing individuals (data not shown), consistent with the hypothesis that the primers amplified more than one target. Two sequences were obtained upon cloning the gmrhg4 PCR product; one sequence corresponded to the gmrhg4 target and the other sequence (GenBank EF644646) contained the polymorphisms observed when sequencing the gmrhg4 PCR product.

We wondered whether an alternative to extensive primer testing would be to eliminate amplification of extraneous targets from the genomic DNA. To remove a target from TILLING assays, sequence information was used to choose a restriction enzyme that cut once within the extraneous target (sequence data from primer testing was sufficient to identify an appropriate enzyme; cloning was not necessary). The restriction-digested DNA was purified by centrifugation through sephadex spin columns prior to performing the TILLING assay. Digestion of the template eliminated amplification of the additional target (Figure 3B) and allowed the identification of 4 more mutant alleles (Additional File 1).

## Discussion

To determine whether soybean is suitable for high-throughput mutation discovery, we screened seven targets in four mutagenized populations and discovered a total of 116 induced mutations. The A and D populations had the highest mutation frequencies, followed by the C and B populations. Given the sequences of the seven targets, the distribution of mutations was as expected. The majority of induced mutations were G/C to A/T transitions. We also found we could discover additional mutations by digesting the template DNA to eliminate an extraneous amplicon that was hampering mutation identification.

Both EMS and NMU mutagenesis of soybean seed resulted in populations with mutation frequencies that are feasible for use in a high-throughput TILLING operation. The mutation frequencies in these soybean populations were higher than those reported for barley and maize [17,19], and except for the B population, are similar or higher than what we have found in our Arabidopsis populations. Although the B population was treated with the same concentration of EMS as the A population, the resulting mutation frequency was lower. It is possible that the genetic background could have an effect on the efficiency or toxicity of the mutagen, as has been observed in rice [21], but differences due to other environmental or experimental conditions cannot be ruled out. The B and C populations are from the same genetic background, but the B population was mutagenized with a 20% lower concentration of EMS and as a result has approximately half the mutation density as the C population. We have noted that treatment of Arabidopsis seed batches with the same concentration of mutagen can vary in mutation frequency from experiment to experiment, probably because of the effect of environmental conditions on the plant response. So it is expected that mutagenesis experiments performed at different locations with different mutagen concentrations may result in very different mutation frequencies. Because soybean is considered a paleopolyploid, it is possible that the mutation frequency could be increased even further without adverse affects due to the genetic redundancy provided by the largely duplicated gene set. For example, allotetraploid and allohexaploid wheat populations have been developed with mutations frequencies of 1/40 and 1/24 Kb, respectively [18]. However, while visible mutations were more frequently observed when the NMU concentration was increased to 3.75 mM, the proportion of treated seeds that germinated and grew was reduced twofold (Ritchie and Nielsen, unpublished observations). Hence, more severe mutation protocols can increase mutation frequency, but they also reduce the recovery of viable seeds dramatically.

In Arabidopsis, maize, and wheat, more than 99% of EMS-induced mutations are G/C to A/T transitions [18-20]. In contrast, the percentage in rice, barley, and Drosophila ranges from 70–84% [17,22,23]. In the EMS-treated soybean populations, the percentage of G/C to A/T transitions was in the range of these previously published frequencies (A = 92%; B = 75%; C = 92%). Based on studies in *E. coli *and mouse [24,25], NMU is also believed to induce primarily G/C to A/T transitions, but few reports are available for plants. Here we find that 90% of mutations induced by NMU were G/C to A/T transitions.

Our study also addressed a problem caused by near identical copies of genes, such as the homeologous sets found in polyploid species or members of gene families. The incompletely sequenced genome makes it difficult to define primers specific for a single gene, so that amplification of multiple products becomes a significant issue for a high-throughput soybean TILLING service. Pre-testing unlabeled primers by amplifying DNA followed by agarose gel electrophoresis and sequencing should reduce the number of primer sets chosen for TILLING that amplify more than one target. We found that pre-testing was successful for soybean targets which are known to be members of gene families (gmclav and gmnark, gmrhg1 and gmrhg4). The maize TILLING service, which faces a similar problem, has successfully implemented such pre-testing in a high-throughput manner [26]. In our study, we found that only ~40% of soybean primers passed pre-testing and of those, only 60% produced high quality TILLING data. Our observation that amplification of multiple products derived from homeologous templates reduces the ability to detect mutations agrees with that of Slade and colleagues [18]. Clearly, robust amplification of a single target will be a requirement for future soybean TILLING. Sequence information from homeologous or paralogous genes could be used to direct primer design toward less conserved regions.

In cases where a primer set that only amplifies one target cannot be identified, it is possible to use sequence information gathered while testing the primers to find a restriction enzyme that digests only one homeolog or paralog thus eliminating amplification from the corresponding template DNA. Restriction digestion adds an extra step and requires larger amounts of template DNA. The step, however, can easily be done in a high throughput manner by digesting templates in 96- or 384-well format prior to PCR and even the additional amount of DNA required would allow at least 1000 genes to be screened with the present DNA yield (1 μg/individual plant).

Legumes have unique biological and agronomic characteristics that cannot be investigated in either Arabidopsis or maize model systems. A TILLING service is currently available for *Lotus japonicus *[27]. While much knowledge will be gained using *L. japonicus *as a model system for legume gene function, the application of that knowledge to modification of soybean traits remains difficult. Given the limits of other functional genomics approaches in soybean [4,28], a TILLING service could provide allelic series in genes of scientific or agronomic importance. Individual mutations may not result in phenotypic changes due to the redundant nature of the soybean genome. However, the high mutation frequency combined with the ability to screen individual targets allows one to screen homeologs or gene family members individually and then combine the mutant alleles through breeding. This would greatly facilitate progress in the study and breeding of soybean and other polyploids in which the efficiency of mutation breeding might otherwise be low. One public service is already operational [29], and others may be developed in the future.

## Conclusion

We have successfully extended the TILLING method to four chemically mutagenized soybean populations in two genetic backgrounds. The substantial mutation density suggests that soybean should be an amenable subject for a high-throughput TILLING service. We have also developed a strategy that could be generally applied to eliminate amplification of multiple products from the soybean genome and it can easily be fit into a high-throughput pipeline.

## Methods

### Mutagenesis and DNA preparation

Soybean (*Glycine max*) seeds were treated with mutagen as detailed in Table 1. For the A population, seeds were soaked in 40 mM EMS for 8 hours followed by 3 washes. EMS was neutralized by 10% sodium thiosulfate solution. For the B population, two sets of 4.5 kg of seeds were imbibed for 9 hours in a solution of 4 L of 40 mM EMS. For the C population, 9 kg of seeds were imbibed for 9 hours in a solution of 8 L of 50 mM EMS. The D population was treated with NMU as detailed by Kerr and Sebastian, except that volumes were reduced by 1/10th [30]. Seeds (2.3 kg) were imbibed in 15 L water for 8 hours with aeration. After draining, the seeds were transferred to 9.8 L of NMU pH 5.5 (buffered with 0.1 M phosphate buffer) for 4 hours with aeration. In all treatments, seeds were rinsed extensively in water prior to planting.

M1 plants were allowed to self-fertilize. Leaf tissue was harvested from the M2 for DNA preparation. DNAs were prepared using commercially available kits; the Fastprep DNA Kit (QBiogene Inc/MP Biomedical, Irvine, CA) as previously described [31], or the DNeasy Plant Kit (Qiagen, Valencia, CA). DNAs were quantitated on 1.5% agarose gels by comparison to Lambda DNA references and normalized for concentration prior to pooling eight-fold.

### PCR primer design

Primers for amplification were designed by entering genomic DNA sequence into the Codons Optimized to Deliver Deleterious Lesions (CODDLe) input form [32] to select the regions most likely to harbor deleterious changes induced by EMS and then using a modified version of Primer3 [33] to select primers.

Following the three initial primers, 27 primer sets were tested for amplification of a single target by agarose gel electrophoresis and sequencing. Of the 27, 17 primer sets amplified more than one target. This was observed in 6 cases on the agarose gel by the appearance of more than 1 molecular weight product, and in 11 cases by sequencing as both products had similar size and could not be distinguished by agarose gel electrophoresis. Only 10 sets of the 27 (37%) amplified one band of the expected size that appeared to consist of a uniform PCR product upon sequencing. Of these 10 primer sets, 4 produced TILLING gels with quality issues such as PCR failure or low yield of PCR product, as well as mispriming. The poor quality of these TILLING gels meant that the primer sets were not appropriate for discovery of induced mutations. However, the remaining 6 of the 10 primer sets produced good quality TILLING gels (see example in Figure 1). These 6 primer sets, plus the initial primer set that was successful, were used to screen all four soybean populations for induced mutations (Table 2).

### High-throughput TILLING

Minor modifications were made to the Arabidopsis TILLING method. Using CODDLe [34], primers were designed to amplify approximately 1.5 kb targets from available sequence. Amplification, CJE digestion, electrophoresis, and sequencing were performed as previously described [20,35] except that 0.15 ng/μl of pooled template was used. The A population was screened in 1-dimensional format while the B, C, and D populations were screened in a 2-dimensional format [36]. In the 1-dimensional format, each sample is present once in a single eight-fold pool per 96-well plate. Pools containing putative mutations must be deconvoluted in a second TILLING assay to identify the mutated individual. In the 2-dimensional format, each sample is present twice in two different eight-fold pools per 96-well plate. The individual containing the putative mutation will be the only sample in common between two pools containing CJE digestion products of the same length. LI-COR 4200 or 4300 (Lincoln, NE) gel images were analyzed using GelBuddy [37].

### Genomic DNA restriction endonuclease digestion followed by high-throughput TILLING

The gmrhg4 PCR product was amplified from the Forrest background and cloned using the pCR 4-TOPO TA kit (Invitrogen, Carlsbad, CA). Both strands of several clones were sequenced to generate consensus sequences for gmrhg4 and the homeolog/paralog. Restriction site differences were found by comparison of the two cloned sequences, and could also be identified by comparison of the gmrhg4 target sequence with the heterozygous sites found when sequencing the gmrhg4 PCR product. Eight-fold pooled DNA samples (4.5 ng total in 5 μl) from the Forrest background were digested for 2 hours at 37°C with 4 units of *ApaI *(NEB, Ipswich, MA) in a volume of 25 μl 1× buffer 4 (NEB). Digests were centrifuged through G-50 medium sephadex (GE Healthcare, Uppsala, Sweden) columns packed in 96-well membrane plates (#MAHVN4550, Fisher Scientific, Pittsburgh, PA) as previously described except that no formamide was added to flow through [31]. 5 μl of flow through was used as template for high-throughput TILLING using primers 5'-cccaaccctaatgtctctccccaaa-3' and 5'-tcccgcagtcaccaacttcacctt-3'. Individual DNAs from a pool were digested with *ApaI *and mixed with *ApaI*-digested wild type DNA to allow detection of homozygous changes. Once individuals were identified, sequencing reactions were performed on the digested templates.

## Authors' contributions

KM, KB, NN, and RR planned and headed the development of the mutant populations. JK, AJ, and SL coordinated the experimental components of the A population development. RL, MD, TE, and SL isolated DNA. BT and JC oversaw the high-throughput laboratory during DNA preparation, arraying, and mutation detection. RL, JC, and MD designed and tested the primers. JC implemented methods for the elimination of multiple amplicons. JC, BT, SH, and LC designed experiments and interpreted the mutation detection data. SH and LC co-directed the high throughput STP laboratory. JC was primarily responsible for drafting and revising the manuscript with contributions from co-authors. All authors read and approved the final manuscript.

## Supplementary Material

Additional File 1 Sequenced nucleotide changes and their predicted effect on the encoded amino acid. Click here for file
