# Supplementary material for: TILLING to detect induced mutations in soybean
Source: BMC Plant Biol. 2008 Jan 24;8:9. doi: 10.1186/1471-2229-8-9 (PMC2266751; doi:10.1186/1471-2229-8-9)
Supplement: Additional File 1 — Sequenced nucleotide changes and their predicted effect on the encoded amino acid. [file 1471-2229-8-9-S1.DOC]

Sequenced nucleotide changes and their predicted effect on the encoded amino acid per target. SIFT^a^ was used to predict whether missense mutations may be deleterious. Changes predicted to be deleterious have SIFT scores of < 0.05. Confident predictions have an IC of < 3.25. Stocks that are likely to be contaminants are indicated by asterisks and were removed from analysis. Stock number prefixes correspond to the populations (A = SB; B = KJ; C = KB; D = RR).

| Target | Sequence | Effect | SIFT (IC) | Zygosity | Individual | Stock |
| --- | --- | --- | --- | --- | --- | --- |
| gmclav | c186y | S81F | 0.00 (3.05) | Hetero | s41F4 | KB_0294 |
|  | c199y | L85= |  | Hetero | s27G4 | RR_0215 |
|  | c301y | A119= |  | Hetero | s29C6 | RR_0395 |
|  | g383a | E147K | 0.95 (3.05) | Homo | s35G8 | RR_0912 |
|  | c426y | S161L | 0.32 (3.05) | Hetero | s37B8 | KB_0058 |
|  | g604a | L220= |  | Homo | s47F7 | KB_0729 |
|  | a626c | N228H | 0.23 (3.05) | Homo | s20A5 | SB1470 |
|  | c677t | L245= |  | Homo | s28C7 | RR_0330 |
|  | c855y | T304I |  | Hetero | s14A6 | SB1012 |
|  | c855y | T304I |  | Hetero | s19H7 | SB1407 |
|  | g860r | E306K | 0.00 (3.05) | Hetero | s29A3 | RR_0362 |
|  | g904r | M320I | 0.26 (3.05) | Hetero | s15F2S | SB1052 |
|  | g927a | R328Q | 0.29 (3.05) | Homo | s14A1 | SB966 |
|  | g979r | Q345= |  | Hetero | s22C2 | SB1635 |
|  | c1081t | I379= |  | Homo | s29G1 | RR_0350 |
|  | t1084w | P380= |  | Hetero | s48H3 | KB_0763 |
|  | c1247y | P435S | 0.00 (3.05) | Hetero | s21F7 | SB1608 |
|  | c1331t | L463F | 0.03 (3.05) | Homo | s29B4 | RR_0371 |
|  | t1357w | T471= |  | Hetero | s55D5 | KJ_0420 |
| gmclavb | c113t | L463F | 0.02 (3.11) | Homo | s29B4 | RR_0371 |
|  | c306t | T527I | 0.25 (3.11) | Homo | s22D6 | SB1680 |
|  | c365y | P547S | 0.00 (3.11) | Hetero | s23E8 | SB1765 |
|  | c403y | F559= |  | Hetero | s23G7 | SB1758 |
|  | c411m | S562* |  | Hetero | s27C8 | RR_0244 |
|  | g516a | G597D | 0.24 (3.17) | Homo | s24F5 | SB1813 |
|  | c521t | Q599* |  | Homo | s47A7 | KB_0724 |
|  | g523r | Q599= |  | Hetero | s56F2 | KY_0462 |
|  | g529t | L601F | 0.06 (3.11) | Homo | s33D6 | RR_0683 |
|  | g691r | G655= |  | Hetero | s26B6 | RR_0146 |
|  | g788a | E688K | 0.04 (3.11) | Homo | s38H6 | KB_0112 |
|  | g903a | G726E | 0.01 (3.11) | Homo | s19H7 | SB1407 |
|  | g944a | G740R | 0.00 (3.11) | Homo | s42B7 | KB_0378 |
|  | g1003r | T759= |  | Hetero | s53G8 | KJ_0319 |
|  | c1006y | N760= |  | Hetero | s31C5 | RR_0532 |
|  | g1021r | E765= |  | Hetero | s17C2 | SB1208 |
|  | g1044a | G773E | 0.00 (3.11) | Homo | s47D5 | KB_0708 |
|  | g1045a | G773= |  | Homo | s28F1 | RR_0255 |
|  | g1048a | E774= |  | Homo | s25B5 | RR_0045 |
|  | g1084a | W786* |  | Homo | s36A6 | RR_0965 |
|  | g1169r | D815N | 0.00 (3.11) | Hetero | s35E8 | RR_0910 |
|  | c1352t | Intron |  | Homo | s28G4 | RR_0292 |
|  | g1466r | R889K | 0.68 (3.10) | Hetero | s56A5 | KJ_0481 |
|  | g1494a | G898= |  | Homo | s30F2 | RR_0431 |
| gmnark | c58y | I416= |  | Hetero | s29H7 | RR_0409 |
|  | c117t | S436F | 0.09 (2.95) | Homo | s35E3 | RR_0824 |
|  | g158a | E450K | 0.25 (2.95) | Homo | s18B5 | SB1305 |
|  | g226a | G472= |  | Homo | s17B2 | SB1207 |
|  | c260y | L484= |  | Hetero | s35C4 | RR_0830 |
|  | c305t | P499S | 0.00 (2.96) | Homo | s19B2 | SB1345 |
|  | c305y | P499S |  | Hetero | s20A4 | SB1454 |
|  | c326y | P506S | 0.49 (2.96) | Hetero | s27H4 | RR_0216 |
|  | g368s | G520R | 0.00 (2.96) | Hetero | s32A2 | RR_0579 |
|  | c377y | P523S | 0.03 (2.96) | Hetero | s26B4 | RR_0117 |
|  | c520y | V570= |  | Hetero | s36D6 | RR_0968 |
|  | c583y | I591= |  | Hetero | s37C1 | KB_0003 |
|  | a620t | S604C | 0.03 (2.96) | Homo | s59B3 | KJ_0658 |
|  | c643y | N611= |  | Hetero | s13C3 | SB908 |
|  | c679y | S623= |  | Hetero | s26E2 | RR_0104 |
|  | g739r | T643= |  | Hetero | s39H2 | KB_0144 |
|  | g768r | G653D | 0.15 (2.96) | Hetero | s44H5 | KB_0501 |
|  | g851r | A681T | 0.04 (2.96) | Hetero | s36B1 | RR_0917 |
|  | g851r | A681T |  | Hetero | s36B5 | RR_0957 |
|  | g853r | A681= |  | Hetero | s19C3 | SB1354 |
|  | g934r | G708= |  | Hetero | s27B5 | RR_0218 |
|  | c937t | I709= |  | Homo | s39B6 | KB_0174 |
|  | g938r | V710M | 0.00 (2.96) | Hetero | s42G7 | KB_0383 |
|  | g1037r | E743K | 0.46 (2.96) | Hetero | s33B7 | RR_0689 |
|  | g1072r | M754I | 0.03 (2.96) | Hetero | s13B1 | SB889 |
|  | g1074r | R755K | 0.13 (2.96) | Hetero | s31B4 | RR_0523 |
|  | c1177t | H789= |  | Homo | s15D4 | SB1066* |
|  | t1243g | H811Q | 0.00 (2.97) | Homo | s15D4 | SB1066* |
|  | c1339t | A843= |  | Homo | s36B6 | RR_0966 |
| gmppck4 | c273y | S31F | 0.00 (2.98) | Hetero | s27G4 | RR_0215 |
|  | c449y | L90F | 0.03 (2.98) | Hetero | s21F5 | SB1585 |
|  | c504t | A108V | 0.00 (2.98) | Homo | s30E4 | RR_0448 |
|  | c598t | F139= |  | Homo | s39B3 | KB_0146 |
|  | g672a | G164E | 0.02 (2.98 | Homo | s41C6 | KB_0307 |
|  | g681r | G167E |  | Hetero | s24B7 | SB1828 |
|  | g681a | G167E | 0.00 (2.98) | Homo | s40G4 | KB_0227 |
|  | g704a | E175K | 0.00 (2.98) | Homo | s36D2 | RR_0935 |
|  | c710t | L177F | 0.01 (2.98) | Homo | s35H2 | RR_0818 |
|  | g856r | P225= |  | Hetero | s27G6 | RR_0231 |
|  | g856r | P225= |  | Homo | s40B6 | KB_0238 |
|  | g886r | A235= |  | Hetero | s56G6 | KJ_0495 |
|  | g934r | R251= |  | Hetero | s32E4 | RR_0604 |
|  | t1076a | F263Y | 0.00 (3.05) | Homo | s18F6 | SB1317 |
| gmrhg1b | t62c | F405= |  | Homo | s41G1 | KB_0269* |
|  | c110t | L421= |  | Homo | s41G1 | KB_0269* |
|  | c122t | V425= |  | Homo | s41G1 | KB_0269* |
|  | c127y | P427L | 0.19 (3.03) | Hetero | s21F2 | SB1555 |
|  | g185a | G446= |  | Homo | s43G5 | KB_0433 |
|  | c329t | V494= |  | Homo | s41G | KB_0269* |
|  | c354y | L503F | 0.64 (3.03) | Hetero | s52H6 | KJ_0240 |
|  | c454y | S536F | 0.26 (3.03) | Hetero | s19D5 | SB1378 |
|  | g463k | W539L | 0.65 (3.03) | Hetero | s32A2 | RR_0579 |
|  | g464c | W539C | 0.18 (3.03) | Homo | s41G | KB_0269* |
|  | g734r | R629= |  | Hetero | s57B1 | KJ_0514 |
|  | t854y | Intron |  | Hetero | s38F4 | KB_0094 |
|  | g1065r | R669= |  | Hetero | s38F4 | KB_0094 |
|  | g1065r | R669= |  | Hetero | s23G5 | SB1741 |
|  | c1159t | L701F | 0.00 (3.03) | Homo | s44C5 | KB_0496 |
|  | c1270t | P738S | 0.00 (3.03) | Homo | s32A4 | RR_0600 |
| gmrhg4^b^ | c516t | T143I |  | Homo | s15B1 | SB_1040 |
|  | a540t | D151V |  | Homo | s23D7 | SB_1755 |
|  | c875y | Q263* |  | Hetero | s15F1 | SB_1044 |
|  | g877a | Q263= |  | Homo | s24G2 | SB_1787 |
|  | g896r | G270R |  | Hetero | s14C5 | SB_1006 |
| gmrhg4b | c41y | L417F | 0.07 (3.15) | Hetero | s22A5 | SB1667 |
|  | g120a | G443D | 0.53 (3.19) | Homo | s28D6 | RR_0321 |
|  | a248: | frameshift |  | Hetero | s51H7 | KJ_0184 |
|  | g340r | Q516= |  | Hetero | s13E4 | SB920 |
|  | c401y | L537F | 0.00 (3.03) | Hetero | s24F7 | SB1834 |
|  | g508r | K572= |  | Hetero | s38F5 | KB_0102 |
|  | g752r | V654I | 0.06 (3.03) | Hetero | s37D3 | KB_0020 |
|  | c995t | Intron |  | Homo | s32H6 | RR_0623 |
|  | g1043a | Intron |  | Homo | s31G1 | RR_0496 |
|  | g1077t | Intron |  | Homo | s25B8 | RR_0078 |
| gmsacpd2 | c44y | V127= |  | Hetero | s43D2 | KB_0404 |
|  | g310a | Intron |  | Homo | s57E6 | KJ_0557 |
|  | g386r | Intron |  | Hetero | s53C6 | KJ_0299 |
|  | c523y | Intron |  | Hetero | s25D4 | RR_0031 |
|  | g610a | Intron |  | Homo | s40E1 | KB_0201 |
|  | g953r | K289= |  | Hetero | s34F1 | RR_0712 |
|  | g1055a | K323= |  | Homo | s36B5 | RR_0957 |
|  | g1087r | G334E | 1.00 (3.09) | Hetero | s52E5 | KJ_0229 |

^a^Ng P and Henikoff S: Predicting deleterious amino acid substitutions. Genome Research 2001, 11(5):863-74

^b^Screening with this primer set used for testing the elimination of multiple amplicons and was not included in calculation of mutation frequency
